# Salivary microbiome changes distinguish response to chemoradiotherapy in patients with oral cancer

**DOI:** 10.1186/s40168-023-01677-w

**Published:** 2023-11-30

**Authors:** Marcell Costa de Medeiros, Stephanie The, Emily Bellile, Nickole Russo, Ligia Schmitd, Erika Danella, Priyanka Singh, Rajat Banerjee, Christine Bassis, George R. Murphy, Maureen A. Sartor, Isabelle Lombaert, Thomas M. Schmidt, Avi Eisbruch, Carol Anne Murdoch-Kinch, Laura Rozek, Gregory T. Wolf, Gen Li, Grace Y. Chen, Nisha J. D’Silva

**Affiliations:** 1 Periodontics and Oral Medicine, University of Michigan School of Dentistry, 1011 North University Ave, Room G018, Ann Arbor, MI 48109-1078 USA; 2 Cancer Data Science Shared Resource, University of Michigan Medical School, 1500 E. Medical Center Dr, Ann Arbor, MI USA; 3 Internal Medicine, University of Michigan Medical School, 1500 East Medical Center Drive, Ann Arbor, MI 331248109 USA; 4 Biologic and Materials Sciences and Prosthodontics, University of Michigan School of Dentistry, 1011 N. University Ave, Ann Arbor, MI USA; 5 Biointerfaces Institute, Ann Arbor, MI USA; 6 Computational Medicine and Bioinformatics, University of Michigan Medical School, 1500 E. Medical Center Dr, Ann Arbor, MI USA; 7 Microbiology and Immunology, University of Michigan Medical School, 1500 E. Medical Center Dr, Ann Arbor, MI USA; 8 Radiation Oncology, University of Michigan Medical School, 1500 E. Medical Center Dr, Ann Arbor, MI USA; 9 Oral Pathology, Medicine and Radiology, Indiana University School of Dentistry, 1011 North Michigan St, Indianapolis, IN USA; 10 Environmental Health Sciences, University of Michigan Medical School, 1500 E. Medical Center Dr, Ann Arbor, MI USA; 11 Otolaryngology, University of Michigan Medical School, 1500 E. Medical Center Dr, Ann Arbor, MI USA; 12 Biostatistics, University of Michigan School of Public Health, University of Michigan Medical School, 1500 E. Medical Center Dr, Ann Arbor, MI USA; 13 Pathology, University of Michigan Medical School, 1500 E. Medical Center Dr, Ann Arbor, MI USA; 14 Rogel Cancer Center, Ann Arbor, MI USA

## Abstract

**Background:**

Oral squamous cell carcinoma (SCC) is associated with oral microbial dysbiosis. In this unique study, we compared pre- to post-treatment salivary microbiome in patients with SCC by 16S rRNA gene sequencing and examined how microbiome changes correlated with the expression of an anti-microbial protein.

**Results:**

Treatment of SCC was associated with a reduction in overall bacterial richness and diversity. There were significant changes in the microbial community structure, including a decrease in the abundance of *Porphyromonaceae* and *Prevotellaceae* and an increase in *Lactobacillaceae*. There were also significant changes in the microbial community structure before and after treatment with chemoradiotherapy, but not with surgery alone. In patients treated with chemoradiotherapy alone, several bacterial populations were differentially abundant between responders and non-responders before and after therapy. Microbiome changes were associated with a change in the expression of DMBT1, an anti-microbial protein in human saliva. Additionally, we found that salivary DMBT1, which increases after treatment, could serve as a post-treatment salivary biomarker that links to microbial changes. Specifically, post-treatment increases in human salivary DMBT1 correlated with increased abundance of *Gemella *spp., *Pasteurellaceae *spp*.*, *Lactobacillus *spp., and *Oribacterium *spp*.* This is the first longitudinal study to investigate treatment-associated changes (chemoradiotherapy and surgery) in the oral microbiome in patients with SCC along with changes in expression of an anti-microbial protein in saliva.

**Conclusions:**

The composition of the oral microbiota may predict treatment responses; salivary DMBT1 may have a role in modulating the oral microbiome in patients with SCC.

**Graphical Abstract:**

After completion of treatment, 6 months after diagnosis, patients had a less diverse and less rich oral microbiome. *Leptotrichia* was a highly prevalent bacteria genus associated with disease. Expression of DMBT1 was higher after treatment and associated with microbiome changes, the most prominent genus being *Gemella*

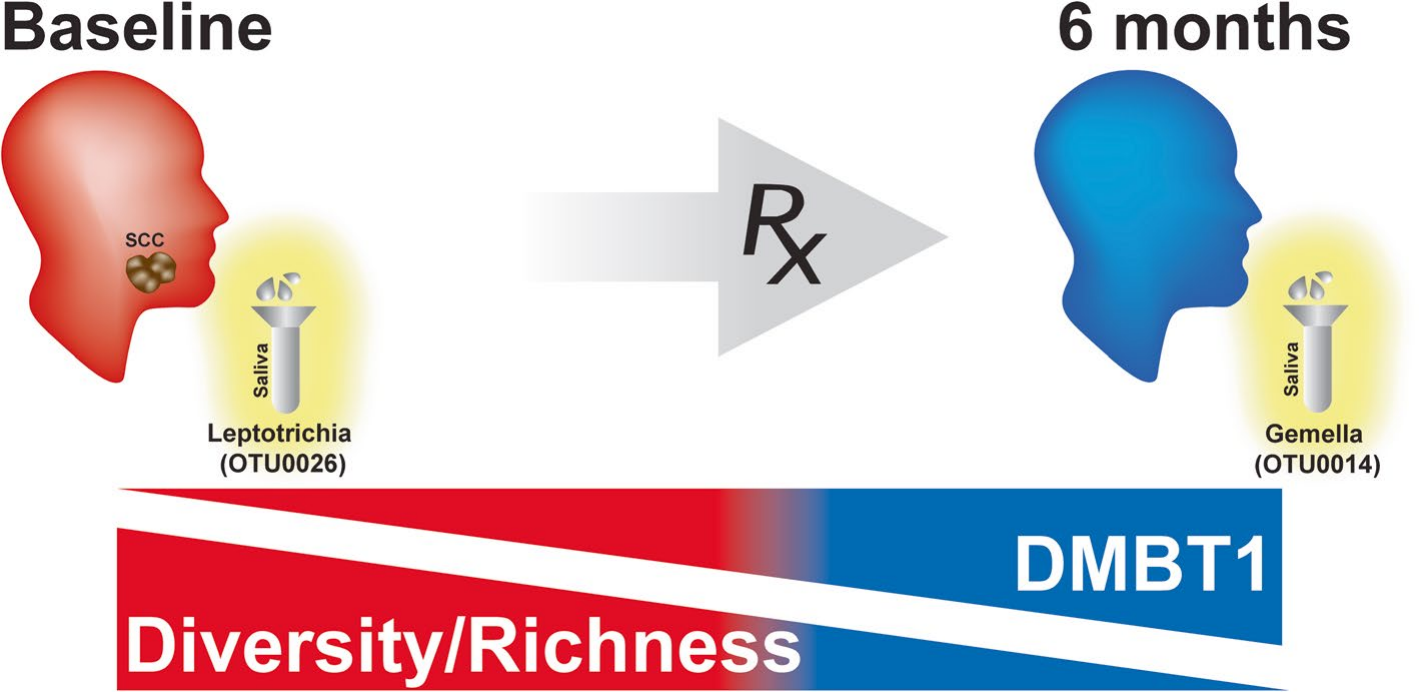

Video Abstract

**Supplementary Information:**

The online version contains supplementary material available at 10.1186/s40168-023-01677-w.

## Background

In the human body, there is approximately one microbial cell for every human cell [1–3]. The majority of microbiota exist along the epithelial lining of the gastrointestinal tract, including the oral cavity, and play important roles in promoting health and disease. Multiple epidemiologic studies identified bacteria associated with cancer development, progression, and response to treatment, suggesting that the microbiome can present diagnostic and prognostic biomarkers [4]. For example, patients with colorectal cancer have an altered gut microbiota compared to that of healthy controls [5]. Both human and mouse studies also demonstrated that gut bacterial composition can impact response to chemotherapy and immune checkpoint inhibitors; in melanoma, commensal bacteria enhanced the anti-tumor efficacy of PD-L1 checkpoint blockade [4]. In particular, *Bifidobacterium *spp., *Bacteroides thetaiotaomicron*, and *B.* *fragilis* increased CTLA-4 response in animal studies [6, 7]. Moreover, several bacteria are associated with the anti-tumor effect of PD-1/PD-L1 inhibitors, among them *Akkermansia*, *Faecalibacterium*, *Clostridiales*, and *Bifidobacterium* spp [4, 8, 9]. When colonized in germ-free mice, bacterial strains that enhanced IFNγ production significantly improved response to immune checkpoint inhibitors and activation of the T cell response [10]. Consistent with a potential role for the gut microbiota in modulating treatment responses, germ-free and antibiotic-treated mice have inferior responses to cancer therapy [7, 11, 12]. Transplantation of fecal microbiota from responders to immune checkpoint inhibitors to tumor-bearing germ-free mice enhanced responses to immune checkpoint therapy [13–15]. Fecal microbiome transplantation also reversed non-responsiveness to immune checkpoint inhibitors in patients with melanoma [16, 17].

The involvement of the microbiome in carcinogenesis and response to treatment at other sites in the gastrointestinal tract, such as the oral cavity, remains understudied [18]. Almost all head and neck cancers are derived from the mucosal epithelial lining of the oral cavity, oropharynx, hypopharynx, or larynx. Squamous cell carcinoma (SCC), the most common head and neck cancer, is the sixth most prevalent cancer worldwide with an incidence of about 600,000 new cases each year [19]. SCC is associated with dysbiosis [2], which is an imbalance in the oral microbiome due to poor oral health [20–22]. More than 700 microbial species, including commensal and opportunistic bacteria, comprise the oral microbiome [23]. Multiple studies have tried to associate specific bacteria and community compositions with SCC to determine causality. Most studies have examined microbial differences between SCC patients and a healthy control group to identify microbial signatures specific for SCC. These studies showed an increase in *Fusobacterium*, *Prevotella*, and *Gemella* species and a decrease in *Streptococcus* and *Rothia* species in SCC [1, 24]. However, due to inter-individual heterogeneity and lack of longitudinal studies examining changes in the oral microbiome within an individual, identification of specific microbes that may be associated with tumor progression or treatment response has been a challenge [22, 25].

Depending on the stage of the disease, patients with SCC are treated with surgery, chemotherapy, and radiation [26]. However, even after appropriate treatment, patients with SCC have an extremely high recurrence rate that contributes to poor survival [27]. Nearly half of all patients treated for SCC develop recurrent or new tumors [27, 28], but repeated tissue biopsies are highly invasive. Therefore, there is a desperate need to improve the prediction of treatment responses. Identifying oral microbial biomarkers that can predict SCC progression and/or treatment responses may significantly improve patient outcomes.

Since saliva is easy to collect and exhibits disease-related changes, multiple studies have explored its diagnostic value. Cross-sectional studies comparing pre-treatment saliva from patients with and without cancer, including SCC and breast cancer, identified cancer-related changes [29–35]. There are several challenges in comparing samples across patients including physiologic and biologic variances [36]. Evaluation of longitudinal samples in a high-risk population could circumvent some of these variances and be highly informative [37, 38]. Longitudinal studies are fewer than cross-sectional studies likely because conventional radiation, used previously to treat SCC, destroys salivary glands, thereby causing xerostomia. Newer therapies, such as intensity-modulated radiotherapy (IMRT), preserve salivary gland function and salivary flow; consequently, potential biomarkers can be assayed over time [39]. For example, in SCC, saliva exhibits changes in protein expression after treatment of SCC [37, 38].

Due to previous challenges with saliva recovery after treatment of SCC, only a few studies have investigated changes in the salivary microbiome after treatment. Those who had surgery showed a prevalence in the salivary microbiome of *Streptococcus* *anginosus*, *Abiotrophia defectiva*, and *Fusobacterium* *nucleatum* at baseline [40]. In an independent study, *Capnocytophaga* and *Leptotrichia* were decreased at 6 months post-surgery [41]. In a cohort of patients who received chemotherapy, the non-responder group was associated with *Fusobacterium* and *Mycoplasma* [42].

Mucosal surfaces have diverse mechanisms to target non-homeostasis-related microorganisms. Deleted in malignant brain tumors 1 (DMBT1), which is highly secreted in saliva, is one such protein with a significant function in epithelial equilibrium, inflammation, innate immunity, and more recently, in the cancer microenvironment [43–45]. In the fluid phase, including saliva, DMBT1 interacts with several bacterial and viral organisms, mainly agglutinating them to facilitate disposal [43, 44]. DMBT1 also interacts with endogenous ligands, like complement pathway components, IgA, lactoferrin, and surfactant proteins [43, 44].

To date, there has been no longitudinal investigation of the salivary microbiome in a large cohort of SCC patients who underwent cancer therapy, including IMRT with and without chemotherapy. We present what we believe is the first study of its kind: a longitudinal investigation of the salivary microbiome in SCC patients before and after treatment of their SCC with comparisons that consider a response to chemoradiotherapy, and changes in the expression of an anti-bacterial protein in saliva. We correlate SCC-related changes in the expression of DMBT1 in saliva with changes in microbiome composition.

## Results

### Longitudinal analysis of salivary microbiome

**Fig. 1:**
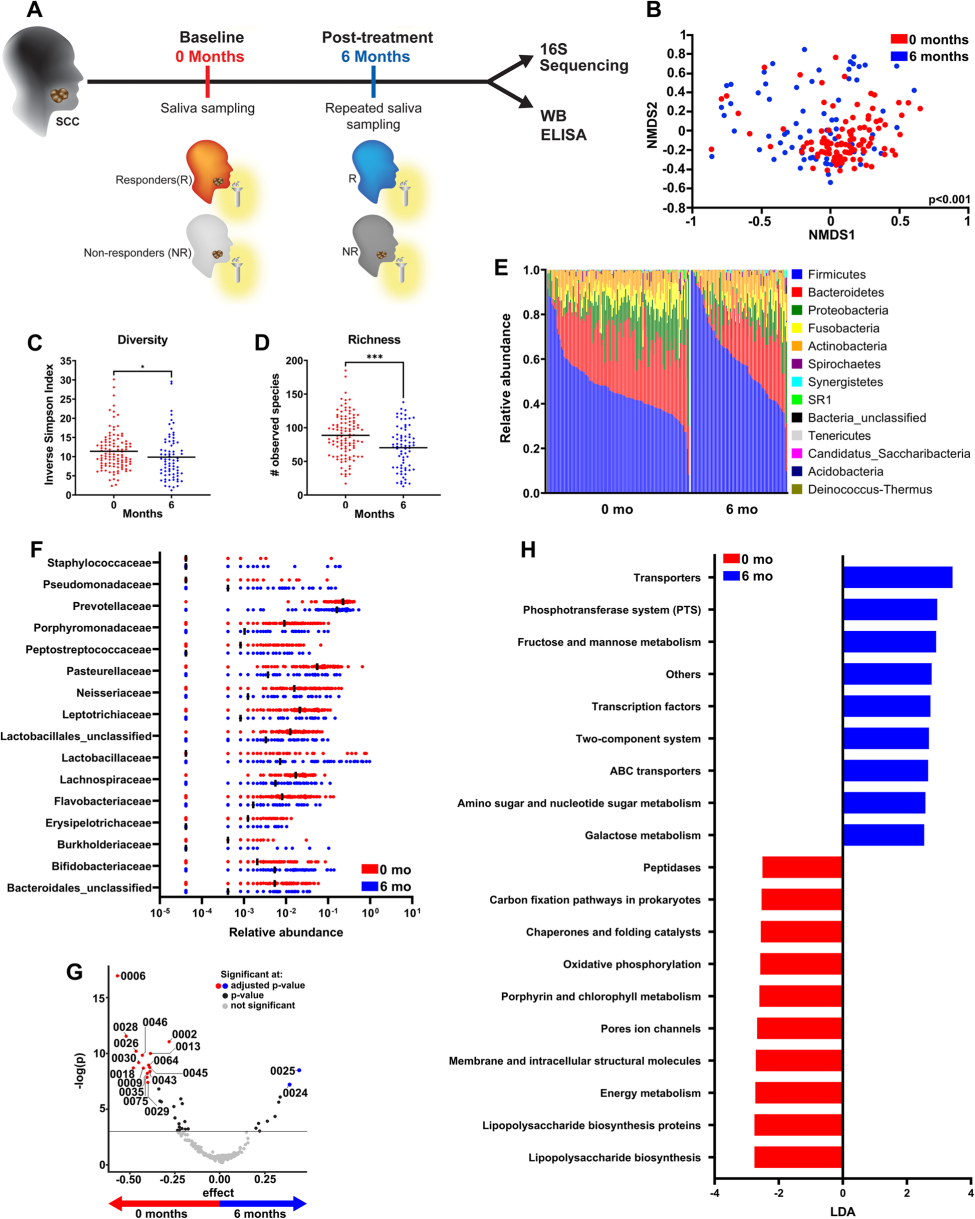
Oral microbiome decreases in diversity and richness after treatment of SCC.** A** Workflow schematic of the entire study, including the sample collection, main methods, and comparisons. **B** Nonmetric multidimensional scaling (NMDS) ordination based on θ_YC_ distances for patients pre- and post-treatment. Diversity (**C**) and richness (**D**) of the salivary microbiome at time 0 (pre-treatment) versus 6 months (post-treatment). **E** Relative abundance of different phyla at 0 and 6 months. **F** Bacterial family members that are > 0.1% in relative abundance and significantly different between pre- and post-treatment saliva (adjusted *p* < 0.05). **G** Volcano plot indicating significantly different OTUs between 0 and 6 months based on ALDEx2 analysis (adjusted *p* < 0.05). **H** Most differentially abundant PICRUSt-predicted KEGG pathways between pre- and post-treatment groups based on LEfSe analysis (LDA cutoff of 2.5). *p < 0.05, ***p < 0.001, and ****p < 0.0001

Figure 1A is a schematic of the entire study workflow including sample collection, analysis, and main comparisons. We investigated changes in the oral microbiome from pre- to post-treatment saliva collected from patients with SCC at diagnosis and 6 months later (0 and 6 months), to identify changes that occurred soon after completion of therapy. Initial treatment consisted of chemoradiotherapy, chemotherapy, radiotherapy, and surgery (Table 1). Non-metric multidimensional scaling (NMDS) analysis demonstrated a significant difference in microbial community structure between 0 and 6 months (Fig. 1B). In addition, both microbial richness and diversity were reduced in post-treatment samples (Fig. 1C, D). There was also a noticeable increase in the phylum Firmicutes (*p* < 0.0001) and decrease in phyla Bacteroidetes (*p* < 0.0001), Fusobacteria (*p* < 0.001), and Proteobacteria (*p* < 0.001) after treatment (Fig. 1E). Among bacterial families that were at least 0.1% in relative abundance, there were significant decreases in *Bacteroidales_unclassified*,* Burkholderiaceae*,* Erysipelotrichaceae*,* Flavobacteriaceae*,* Lachnospiraceae*,* Neisseriaceae*,* Lactobacillales_unclassified*,* Leptotrichaceae*,* Prevotellaceae*,* Pasteurellaceae*,* Peptostreptococcaceae*, and *Porphyromonadaceae,* and noticeable increases in *Bifidobacteriaceae*, *Lactobacillaceae*,* and Pseudomonadaceae* (Fig. 1F). There were several OTUs that were significantly decreased after treatment including unclassified *Pasteurellaceae *spp. (OTU0006), *Porphyromonas* (OTU0028), *Leptotrichia* (OTU0026), *Prevotella* (OTU0018), *Leptotrichia* (OTU0030), *Oribacterium* (OTU0046), *Neisseria* (OTU0009), unclassified *Bacteroidales *spp. (OTU0035), *Leptotrichia* (OTU0075), *Prevotella* (OTU0029), *Capnocytophaga* (OTU0043), *Lachnoanaerobaculum* (OTU0045), unclassified *Flavobacteriaceae *spp. (OTU0064), unclassified *Lactobacillales *spp. (OTU0013), and *Prevotella* (OTU0002) (Fig. 1G, ALDEx2 method with Benjamini–Hochberg adjusted p-value and Supplementary Fig. 1A). There were only two OTUs from this analysis that were significantly increased after treatment, that being *Streptococcus* (OTU0024) and *Lactobacillus* (OTU0025).

**Table 1:** Demographics and clinical characteristics (Figs. 1, 2 and 3)

**Variable**		**0-month cohort ***N* = 106	**6-month cohort ***N* = 72	**Chemorad paired 0- and 6-month cohort ***n* = 33	**Surgery paired 0- and 6-month cohort ***n* = 15	**All HNSCC ***n* = 109
**Age**	Years	58.5 (9.5)	57.3 (9.6)	59.8 (8.4)	53.1 (9.0)	58.3 (10.0)
**Gender**	Male	82 (77%)	54 (75%)	25 (76%)	11 (73%)	85 (78%)
	Female	24	18	8	4	24
**Clinical Stage**	0/1	13	8	-	7	13
	2	9	7	1	-	9
	3	16	12	5	3	17
	4	68 (64%)	45 (63%)	27 (82%)	5 (33%)	70 (64%)
**T stage**	T1	32	23	6	11	33
	T2	25	19	11	1	26
	T3	18	12	7	2	18
	T4	28	16	8	1	29
	X	3	2	1	-	3
**N stage**	N0	40	26	3	11	40
	N1	8	5	3	-	10
	N2	53	39	27	4	54
	N3	5	2	-	-	5
**M stage**	M1	1	1	-	1	1
**Disease Site**	Larynx	26	13	4	3	26
	Oral Cavity	21	17	1	7	22
	Oropharynx	53	38	27	5	55
	Nasopharynx	1	1	-	-	1
	Hypopharynx	2	2	-	-	2
	Unknown primary	3	1	1	-	3
**Initial Treatment**	Chemoradiotherapy	50	33	33	-	52
	Chemotherapy	2	1	-	-	2
	Radiotherapy	7	4	-	-	7
	Surgery	43	30	-	15	44
	Unknown	4	4	-	-	4
**ACE Comorbidities Score**	None	31	23	10	4	32
	Mild	52	32	17	8	54
	Moderate	16	12	5	3	16
	Severe	7	5	1	-	7
**BMI**	Underweight (< 18.5)	3	2	-	-	3
	Normal (15.5–24.9)	31	19	6	6	32
	Overweight (25–29.9)	41	30	15	6	42
	Obese (30 +)	31	21	12	3	32
**HPV status (among OP only)**	Positive	42 (79%)	31 (82%)	22 (81%)	3 (60%)	44 (80%)
**Drinker**	Never	5	4	1	1	5
	Current	77	56	25	12	80
	Former (quit > 12 months)	24	12	7	2	24
**Smoker (cigarettes)**	Never	26	19	7	4	27
	Current	46	36	14	9	47
	Former (quit > 12 months)	34	17	12	2	35
**First Recurrence Pattern**	Persistent Disease	3	2	0	1	3
	Locoregional	7	4	1	2	8
	Distant	6	4	3	-	6
	Locoregional + Distant	6	6	4	-	6

Some of these were also found to be most differentially abundant between 0 and 6 months by Linear discriminant analysis Effect Size (LEfSe) analysis, although additional OTUs were found to be associated with baseline samples, including *Actinomyces* (OTU0007) and *Prevotella* (OTU0010), or enriched in post-treatment samples, including *Lactobacillus* (OTU0011), *Prevotella* (OTU0019), *Lactobacillus* (OTU0020), and *Pseudomonas* (OTU0066) (Supplementary Fig. 1B). Altogether, these studies show that the salivary microbiome changes after treatment of SCC.

The oral microbiome also participates in the host metabolic system, maintaining host immune system homeostasis and protecting against pathogen colonization [46, 47]. Therefore, Phylogenetic Investigation of Communities by Reconstruction of Unobserved States (PICRUSt) analysis was used to infer functional categories associated with taxonomy composition, and predicted pathways were classified using the Kyoto Encyclopedia of Genes and Genomes (KEGG) database. Interestingly, biofunctional pathways that were enriched in SCC patients before treatment were related to membrane and intracellular structural molecules and lipopolysaccharide biosynthesis, which may reflect bacterial pathogenicity, toxicity, and anti-microbial resistance (Fig. 1H, red bars). After treatment, there was an enrichment of pathways related to transporters, phosphotransferase system (PTS), and sugar metabolism (Fig. 1H, blue bars).

### Salivary microbiome profiles associated with surgery alone or chemoradiotherapy

**Fig. 2:**
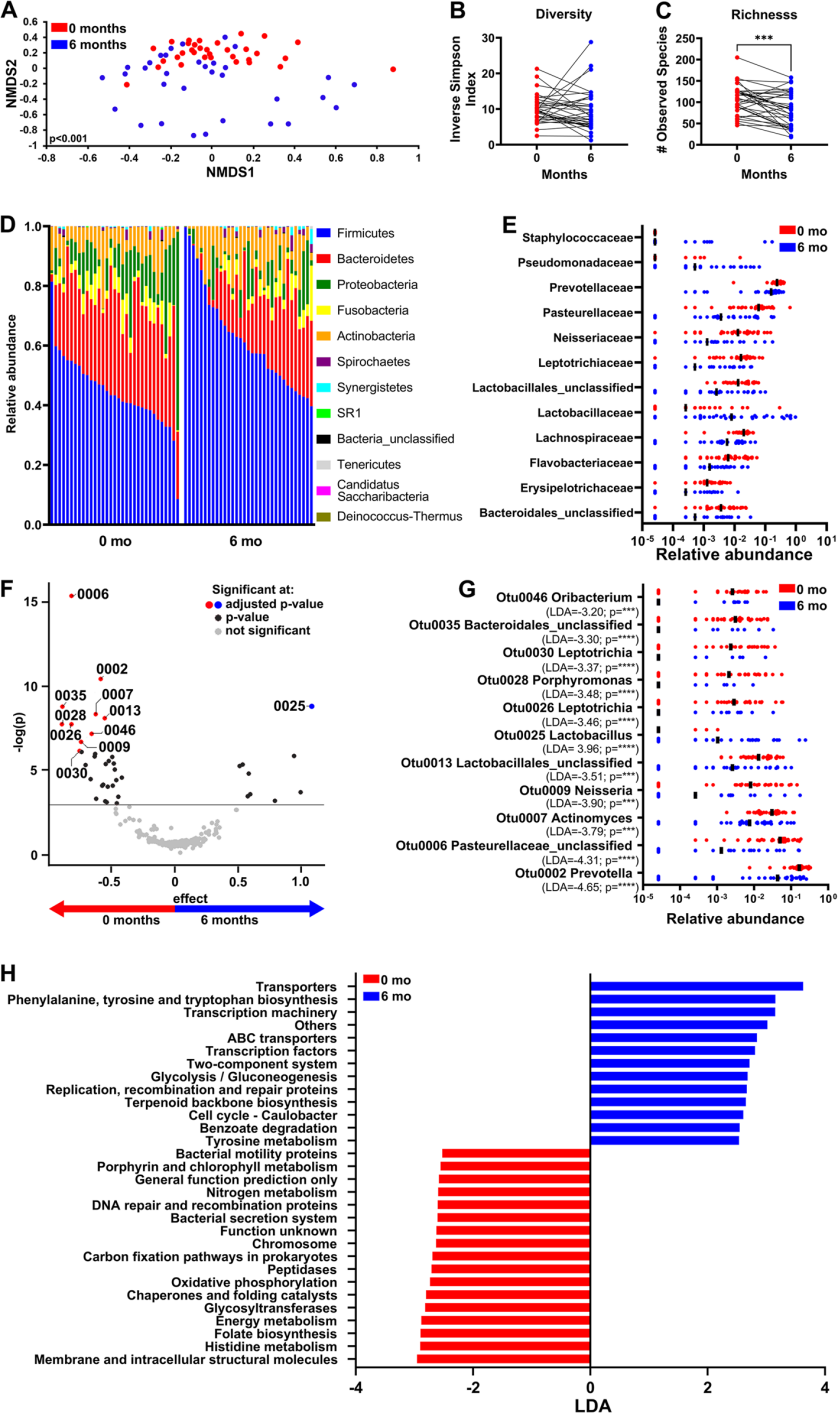
Significant changes in microbiome after chemoradiotherapy. **A** NMDS ordination plot showing community structure differences (β-diversity), diversity (**B**), and richness (**C**) of chemoradiotherapy-treated SCC patients at 0 and 6 months. **D** Relative abundance of bacteria at the phylum level. **E** Bacterial families that are > 0.1% in abundance and significantly different (adjusted *p* < 0.05) before and after chemoradiotherapy. **F** Differentially abundant OTUs at 0 and 6 months post-chemoradiotherapy based on ALDEx2 data analysis (adjusted *p* < 0.05). **G** Relative abundance of significantly different OTUs identified by ALDEx2 and LEfSe analysis. **H** PICRUSt-predicted KEGG pathways that are most differentially abundant between pre- and post-chemoradiotherapy samples based on LEfSe analysis (LDA cutoff of 2.5) *p < 0.05, ***p < 0.001, and ****p < 0.0001

**Fig. 3:**
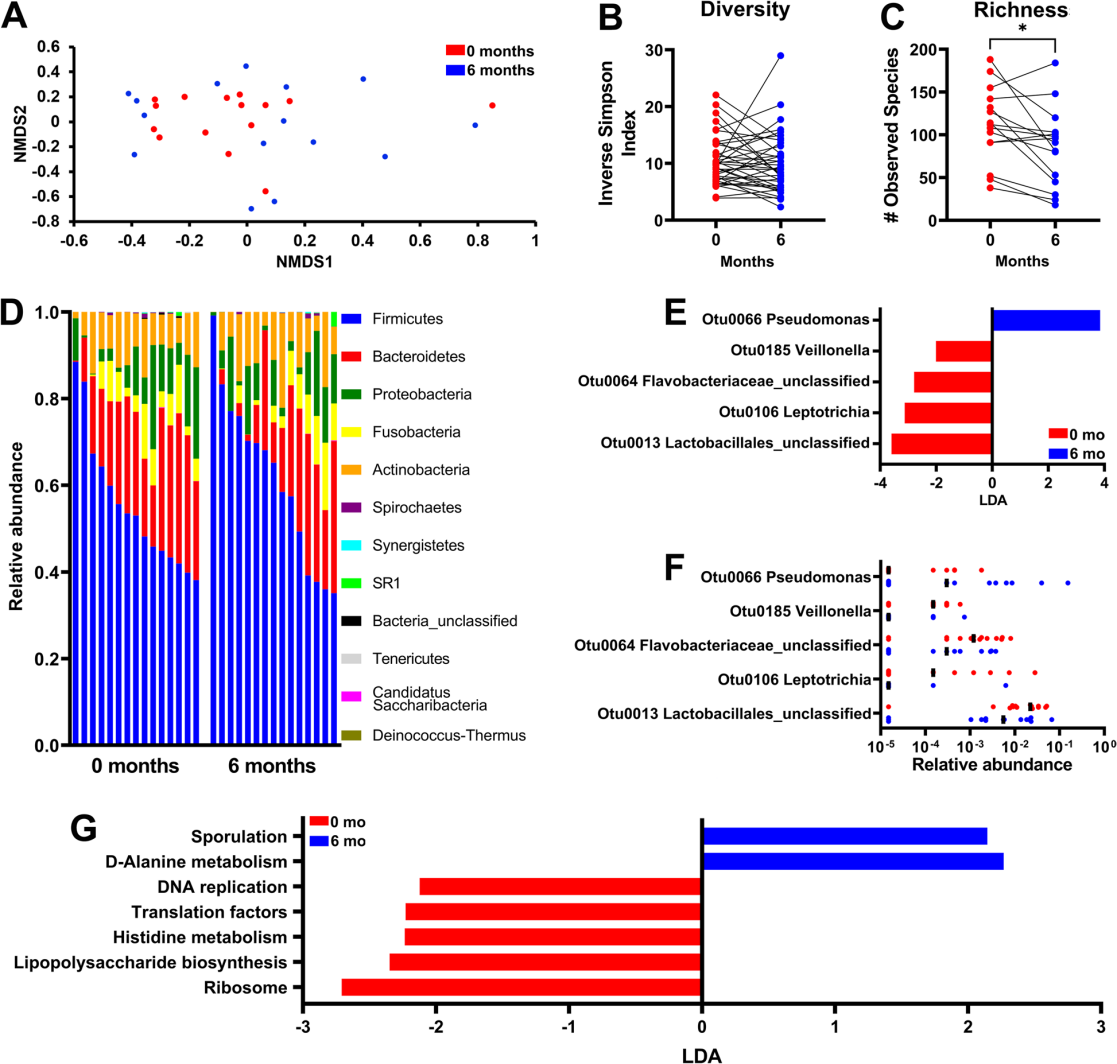
Significant change in the richness of the salivary microbiome after surgery alone. **A** β-diversity shown by NMDS plot, (**B**) diversity, and (**C**) richness of the salivary microbiome at 0 and 6 months in SCC patients treated with surgery alone. **D** Relative abundance of salivary bacteria at 0 and 6 months at the phylum level. **E** Most differentially abundant OTUs between pre- and post-surgery salivary microbiomes based on LEfSe analysis and (**F**) their relative abundances. **G** Most differentially abundant PICRUSt-predicted KEGG pathways before and after surgery. *p < 0.05

Radiotherapy, surgery, chemoradiotherapy, and chemotherapy are treatment options for SCC [28]. Since the microbiome can influence treatment responses [14], we investigated microbial changes associated with either chemoradiotherapy (Fig. 2) or surgery (Fig. 3) in saliva using matched samples collected at time 0 and 6 months from SCC patients. Patients who underwent surgery followed by adjuvant radiation, surgery followed by chemoradiation or who received only radiotherapy or only chemotherapy were excluded due to small sample sizes (< 12/group).

Patients who had undergone chemoradiotherapy with matched time 0- and 6-month samples (*n* = 33) exhibited a significant difference in microbial community structure at 6 months compared to time 0 before initiation of treatment (Fig. 2A). No significant change in diversity was noted (Fig. 2B) although richness decreased after treatment (Fig. 2C). At the phylum level, there was an increase in the relative abundance of Firmicutes (*p* < 0.0001) and decrease in the relative abundance of Bacteroidetes (*p* < 0.0001) and Proteobacteria (*p* < 0.05) (Fig. 2D). In addition, of the families that were > 0.1% in abundance, there was a significant decrease in *Prevotellaceae*,* Pasteurellaceae*,* Neisseriaceae*,* Leptotrichiaceae*, and *Lachnospiraceae* and increase in *Lactobacillaceae* and *Pseudomonadaceae* families after chemoradiation (Fig. 2E). OTUs associated with pre- and post-treatment were further identified by ALDEx2 [48]. Specifically, there were significant increases in *Lactobacillus* (OTU0025) and decreases in unclassified *Pasteurellaceae *spp. (OTU0006), *Bacteriodales* (OTU0035),* Porphyromonas* (OTU0028), *Leptotrichia* (OTU0026), and* Prevotella* (OTU0002) (Fig. 2F, G). Some of these OTUs were also identified as differentially abundant before and after chemoradiotherapy by LEfSe analysis (Supplementary Fig. 2). PICRUSt analysis showed a higher pre-treatment association of pathways involved in membrane and intracellular structural molecules, energy metabolism, bacterial secretion system, and bacterial motility proteins; increased representation of pathways involved in transporters and phenylalanine, tyrosine, and tryptophan biosynthesis was associated with post-treatment (Fig. 2H). Together, these studies showed that the salivary microbiome changes after treatment of SCC with chemoradiotherapy.

For patients treated with surgery alone (*n* = 15), no significant differences were noted in overall community structure (Fig. 3A) and diversity (Fig. 3B). Interestingly, richness was significantly reduced at 6 months (Fig. 3C). Although there were no significant differences in relative abundance on phyla (Fig. 3D) or family level (data not shown) before and after surgery, LEfSe analysis showed pre-treatment predominance of unclassified *Lactobacillales *spp. (OTU0013), *Leptotrichia* (OTU0106), unclassified *Flavobacteriaceae *spp. (OTU0064), and *Veillonella* (OTU0185) and *Pseudomonas* (OTU0066) after treatment (Fig. 3E, F).

KEGG pathways that correlated with treatment based on PICRUSt and LEfSe analysis showed increased representation of pathways related to genetic information processing, such as translation (i.e., ribosome), lipopolysaccharide biosynthesis, and DNA replication prior to surgery and pathways involving amino acid metabolism (D-Alanine) after treatment (Fig. 3G).

### Changes in salivary microbiota associated with response to chemoradiotherapy

Chemoradiotherapy is often given definitively in lieu of surgery, and therefore identifying biomarkers indicative of response has significant clinical implications. Saliva samples collected from patients pre- (*n* = 50) and post-chemoradiotherapy (*n* = 33) were used to identify potential microbial biomarkers associated with response to chemoradiotherapy. Patients were deemed non-responders if any recurrence (local or metastatic) occurred within the follow-up period (*n* = 11) whereas responders were considered disease-free until the time of longest follow-up (up to ~ 5 years, *n* = 39 (Table 2).

**Table 2:** Demographics distribution by chemoradiotherapy response (Figs. 4 and 5)

**Variable**		**Responders (*n* = 39)**	**Non-responders (*n* = 11)**	
		*n* or mean (std)	*n* or mean (std)	***p* value**
**Age**	Years	58.5 (7.6)	61.7 (9.9)	0.26
**Gender**	Male	32	8	0.49
	Female	7	3	
**Clinical stage**	0/1	-	-	0.34
	2	1	1	
	3	3	2	
	4	35	8	
**T stage**	T1	5	1	0.95
	T2	11	4	
	T3	9	2	
	T4	13	4	
	X	1	-	
**N stage**	N0	3	3	0.31
	N1	3	1	
	N2	31	7	
	N3	2	-	
**Disease site**	Larynx	7	3	0.21
	Oral cavity	-	1	
	Oropharynx	31	7	
	Unknown primary	1	-	
**ACE comorbidities Score**	None	12	3	0.79
	Mild	20	5	
	Moderate	6	2	
	Severe	1	1	
**BMI**	Normal (15.5–24.9)	7	4	0.34
	Overweight (25–29.9)	18	5	
	Obese (30 +)	14	2	
**HPV status**	Negative	7	6	0.04
	Positive	29	4	
	Unknown	3	1	
**Drinker**	Never	-	1	0.16
	Current	28	7	
	Former (quit > 12 months)	11	3	
**Smoker (cigarettes)**	Never	11	-	0.14
	Current	16	6	
	Former (quit > 12 months)	12	5	

**Fig. 4:**
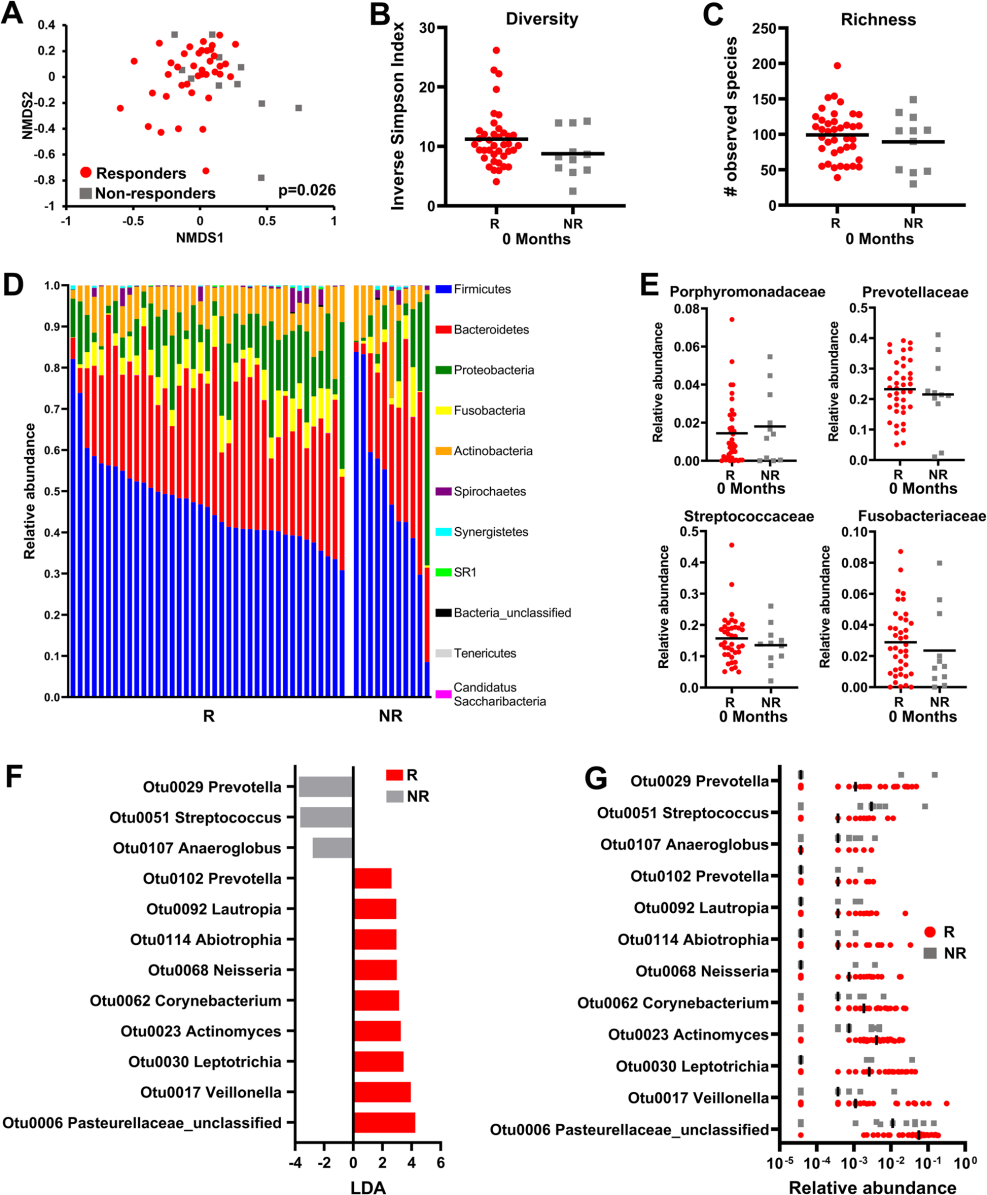
*Prevotella* is associated with non-responders to chemoradiotherapy at baseline.** A** β-diversity shown by NMDS plot, (**B**) diversity, and (**C**) richness of the salivary microbiome sampled before treatment in SCC patients that were responders (R) (i.e., no local or distant recurrences) versus non-responders (NR) to chemoradiotherapy. **D** Relative abundance of salivary bacteria at the phylum level between responders and non-responders at baseline. **E** Relative abundance of the bacterial families *Porphyromonadaceae*, *Prevotellaceae*, *Streptococcocaceae*, and *Fusobacteriaceae*. **F** LEfSe analysis showing the most differentially abundant OTUs at baseline between responders versus non-responders and (**G**) their relative abundances

At baseline (0 months), θ_YC_ distances between bacterial communities revealed a significant difference in overall bacterial profile (Fig. 4A) but no difference in diversity (Fig. 4B) and richness (Fig. 4C), between responders and non-responders. Both responders and non-responders had similar relative abundances of bacteria belonging to the different phyla prior to starting chemoradiotherapy (Fig. 4D). Additionally, there were no significant differences in the relative abundance of any of the observed bacterial families that are greater than 0.1% in abundance within the saliva between non-responders and responders (data not shown), including families that have been previously associated with oral cancer [49] such as *Porphyromonadaceae*, *Prevotellaceae, Streptococcaceae,* or *Fusobacteriaceae* (Fig. 4E). Although ALDEx2 analysis did not predict any OTUs at baseline that were significantly associated with treatment response, LEfSe analysis of baseline salivary samples showed a significant association between non-responders and *Prevotella* (OTU0029) (Fig. 4F)*,* whereas unclassified *Pasteurellaceae *spp. (OTU0006), *Veillonella* (OTU0017), *Leptotrichia* (OTU0030), *Corynebacterium* (OTU0062), and *Lautropia* (OTU0092) were more abundant in responders (Fig. 4G). PICRUSt analysis did not reveal any predicted functional pathways associated with response or recurrence (data not shown).

**Fig. 5:**
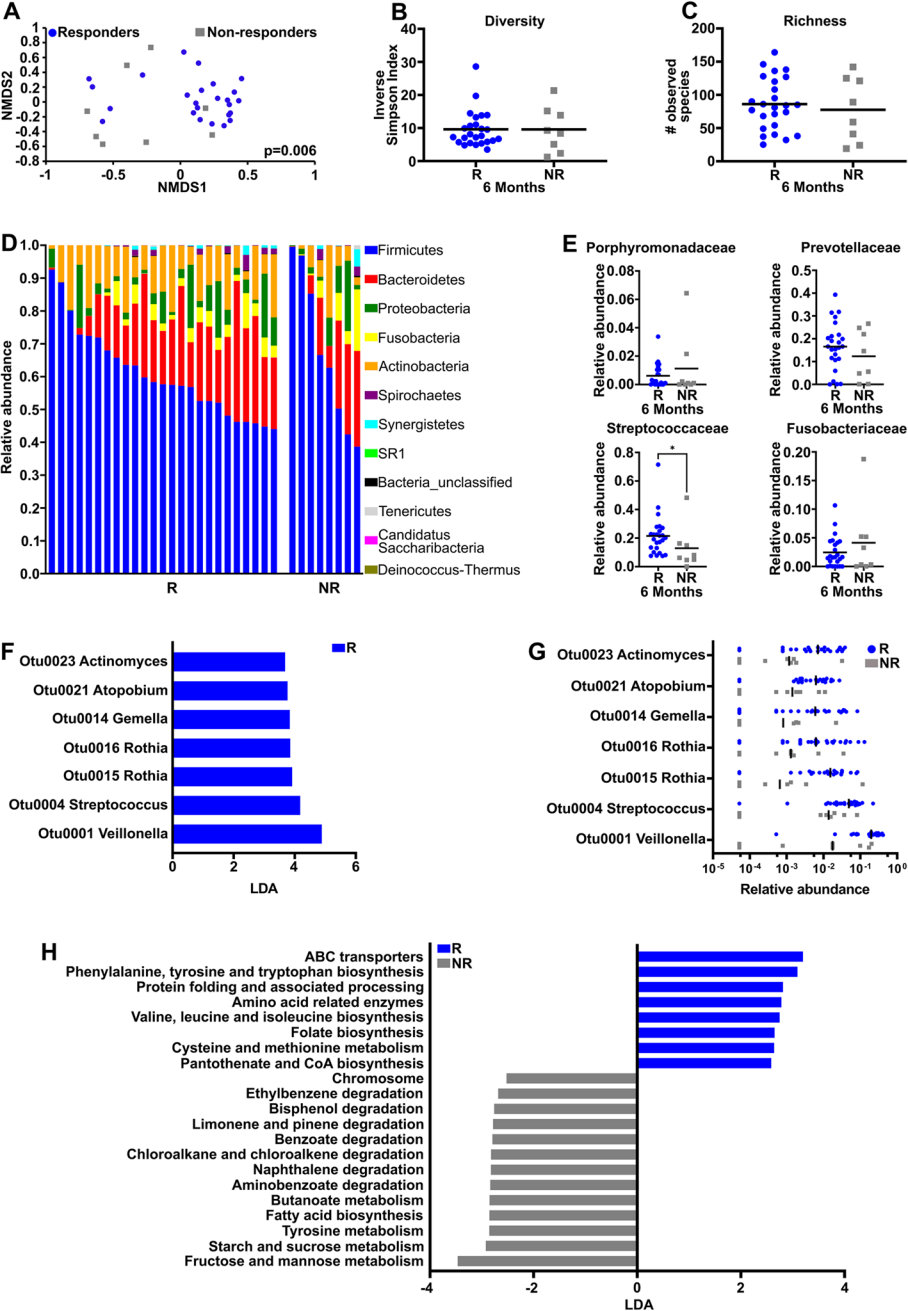
Microbiome differences between responders and non-responders to chemoradiotherapy at 6 months.** A** NMDS plot comparing responders (R) vs non-responders (NR) after treatment. Diversity (**B**) and Richness (**C**) plots. **D** Phylogenetic composition at the phylum level in saliva samples based on treatment response after chemoradiotherapy. **E** Relative abundance of *Porphyromonadaceae*, *Prevotellaceae*, *Streptococcaceae*, and *Fusobacteriaceae*. **F** LEfSe analysis identifying the most differentially abundant OTUs between responders and non-responders after chemoradiotherapy. **G** Relative abundance of OTUs as identified by LEfSe (LDA > 3.5). **H** Most differentially abundant PICRUSt-predicted KEGG pathways in the salivary microbiome of responders and non-responders after chemoradiotherapy. *p < 0.05

When comparing the salivary microbiota between available non-responders (*n* = 8) and responders (*n* = 25) after completion of treatment at 6 months from baseline, there was a higher significant difference (*p* = 0.006) in the overall community structure based on θ_YC_ distances (Fig. 5A), but no differences in overall diversity (Fig. 5B) or richness (Fig. 5C). Both responders and non-responders also had similar abundances of bacteria in the Firmicutes, Bacteroidetes, and Fusobacteria phyla post-treatment (Fig. 5D), and as with pre-treatment salivary microbiota in responders versus non-responders, there were no significant differences in the relative abundance of the different bacterial families that are typically associated with oral cancer dysbiosis, including *Porphyromonadaceae*, *Prevotellaceae,* or *Fusobacteriaceae.* However, a significant decrease in *Streptococcaceae* was observed (Fig. 5E and data not shown). LEfSe analysis of post-treatment samples showed a significant association between responders and the relative abundance of *Veillonella* (OTU0001), *Streptococcus* (OTU0004), *Rothia* (OTU0015 and OTU0016), *Gemella* (OTU0014), *Atopobium* (OTU0021), and *Actinomyces* (OTU0023) (Fig. 5F, G).

Since microbial metabolites can affect response to therapy [50], PICRUSt was performed to predict functional pathways inferred from 16 s rRNA sequences that could potentially be associated with response. Unlike pre-treatment samples that showed no difference in predicted metabolic pathways between responders and non-responders (data not shown), post-treatment samples showed that responders had an increased representation of pathways associated with amino acid biosynthesis and metabolism (Fig. 5H). Non-responders, on the other hand, had an increase in multiple pathways involved in sugar metabolism, tyrosine metabolism, and as well as in fatty acid biosynthesis (Fig. 5H).

### SCC downregulates DMBT1 in saliva

**Fig. 6:**
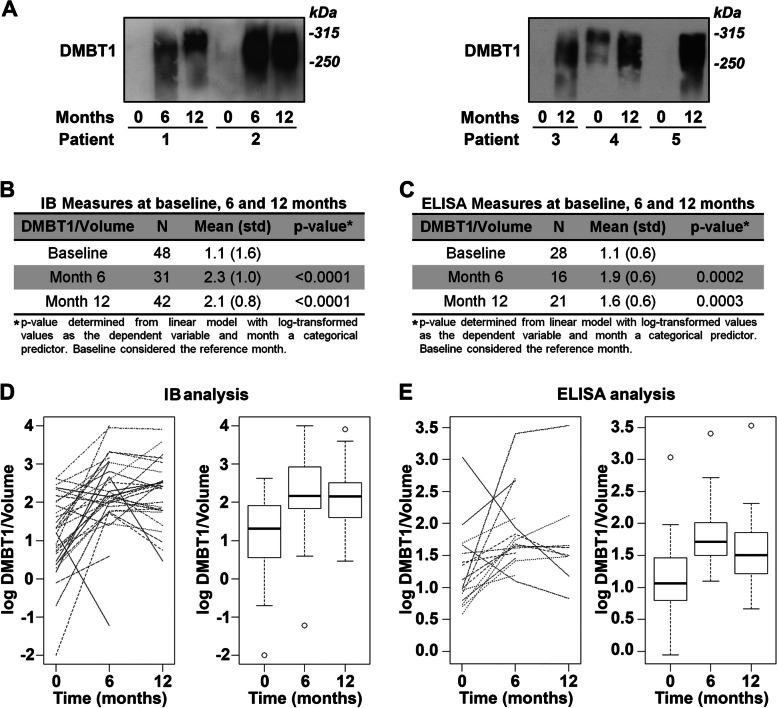
DMBT1 secretion is suppressed in saliva from untreated SCC patients. DMBT1 levels were analyzed at baseline and 6 and 12 months for each patient. **A** Representative immunoblots of DMBT1 in saliva samples normalized to sample volume. **B**, **C** Log-transformed values. *P* values were determined using linear mixed models with compound symmetric variance structure assumed and baseline as a reference category. **B** Densitometric quantification of immunoblot data normalized to sample volume. **C** DMBT1 levels in saliva samples as determined by ELISA. **D**, **E** Each DMBT1 measure from immunoblot and ELISA quantification, respectively, was log-transformed

We previously demonstrated that DMBT1, an anti-microbial protein, is downregulated in SCC and is associated with increased invasive capacity and poor prognosis [44]. DMBT1 is strongly expressed in the salivary gland and constitutes up to 10% of secreted protein in saliva [43, 51–53]. To determine whether the expression of DMBT1 in saliva from SCC patients changed with treatment, immunoblot analysis (Fig. 6A) was performed on a subset of patients (Table 3). Immunoblot analysis of saliva samples from 48 patients with SCC revealed low DMBT1 expression prior to treatment and significantly increased levels at 6 (2.1 fold average) and 12 months (1.9 fold) after treatment, compared with baseline in volume-normalized samples (Fig. 6B, D). Similar findings were observed by ELISA (Fig. 6C, E) on saliva samples from a subset (*n* = 28) of the 48 patients screened by immunoblot analysis. These data show that DMBT1 expression is increased after treatment of SCC regardless of treatment regimen.

**Table 3:** Demographics distribution for DMBT1 expression study (Fig. 6)

**Variable**		**DMBT1 expression cohort**
		*n* = 48
**Age**	Years	57.0 (9.4)
**Gender**	Male	40 (83%)
	Female	8
**Clinical stage**	0/1	1
	2	3
	3	5
	4	36 (75%)
	Unknown	3
**T stage**	T1	7
	T2	20 (41%)
	T3	5
	T4	13
	Unknown	3
**N stage**	N0	8
	N1	5
	N2	30 (62%)
	N3	5
**Disease site**	Larynx	6
	Oral cavity	8
	Oropharynx	29 (60%)
	Nasopharynx	1
	Hypopharynx	1
	Unknown primary	3
**Initial treatment**	Chemoradiation	31 (64%)
	Chemo alone	1
	Radiation alone	1
	Surgery	12
	unknown	3
**ACE comorbidities score**	None	14
	Mild	24 (50%)
	Moderate	5
	Severe	2
	Unknown	3
**BMI**	Underweight (< 18.5)	1
	Normal (15.5–24.9)	9
	Overweight (25–29.9)	21 (44%)
	Obese (30 +)	14
	Unknown	3
**HPV status**	Positive	28 (58%)
	Negative	17
	Unknown	3
**Drinker**	Never	1
	Current	37
	Former (quit > 12 months)	7
	Unknown	3
**Smoker (cigarettes)**	Never	13
	Current	17
	Former (quit > 12 months)	15
	Unknown	3

**Fig. 7:**
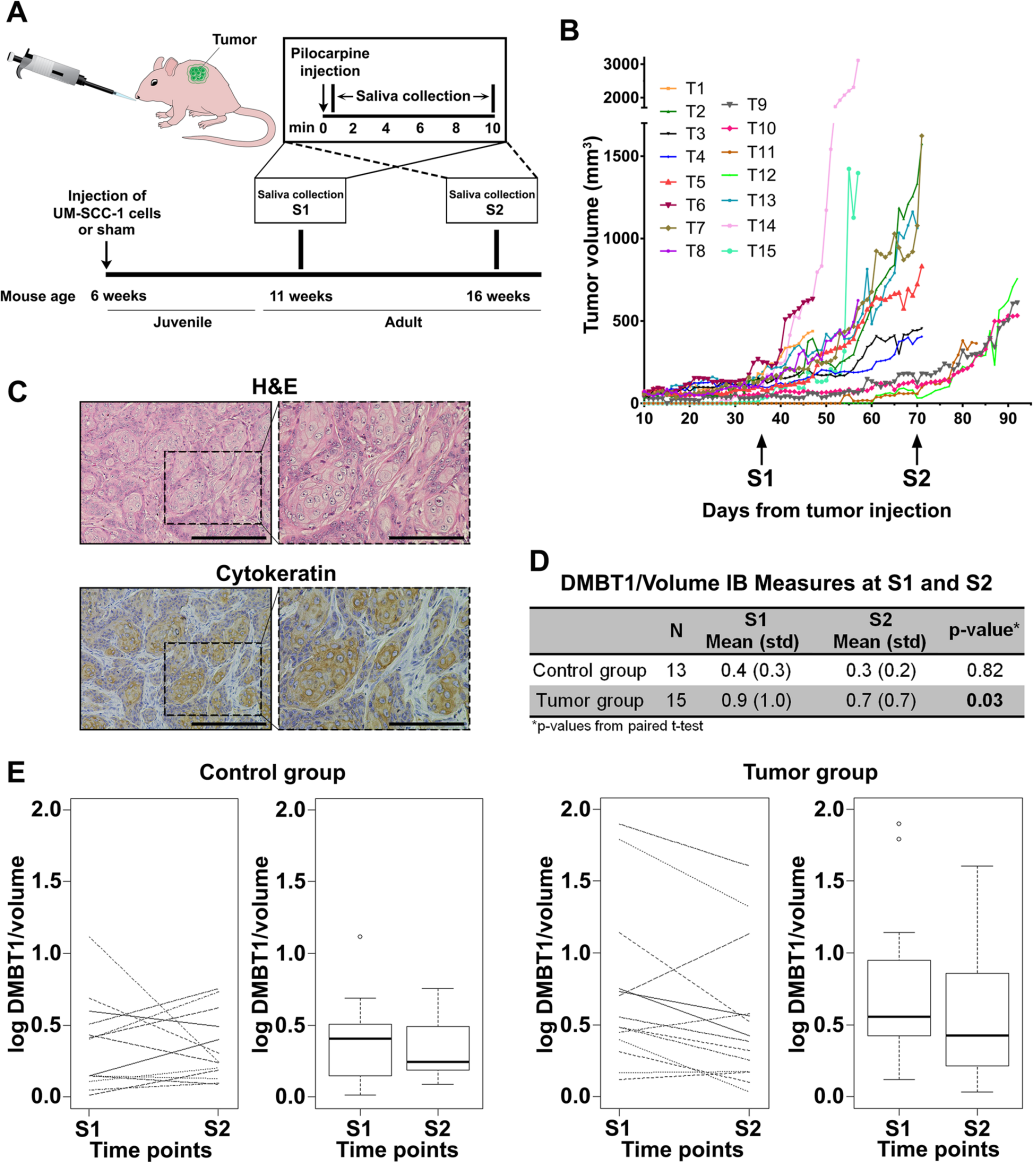
Salivary DMBT1 is reduced in mice after tumor development.** A** Schematic showing the timing of saliva collection. UM-SCC-1 cells or matrigel (control) were injected subcutaneously into athymic nude mice and whole stimulated saliva was collected. **B** Tumor volume was measured for 60 days. **C** Representative tumor section stained with hematoxylin–eosin and cytokeratin antibody. Scale bar = 500 µm in the left panel and 200 µm in the right panel. **D** Densitometric quantification of immunoblot data normalized to saliva volume collected at two time points (S1 and S2) in each adult mouse and differences tested by paired *t* test. **E** Each DMBT1 measure from immunoblot quantification was log-transformed

To directly investigate the extent to which SCC modulates DMBT1 secretion in saliva, UM-SCC-1 cells were injected subcutaneously into mice, and DMBT1 was quantified in saliva from adult mice at two time points (Fig. 7A). DMBT1 was normalized to saliva volume to accommodate variations in secretion between mice. Starting 10 days after injection, tumor size was measured and tumor volume calculated (Fig. 7B). The presence of SCC was verified by hematoxylin–eosin stain and cytokeratin immunohistochemistry (Fig. 7C). There was a significant decrease in DMBT1 secretion in adult mice with tumors (paired *t* test, *p* = 0.03) whereas DMBT1 secretion in control mice was not significantly different between the two time points (*p* = 0.82) (Fig. 7D, E). The interaction term for group x time in a linear mixed model for this experiment trended toward significance (*p*=0.11). Together both human and mouse saliva studies show that SCC suppresses DMBT1 expression in saliva.

### DMBT1 levels in saliva correlate with specific bacterial populations

**Fig. 8:**
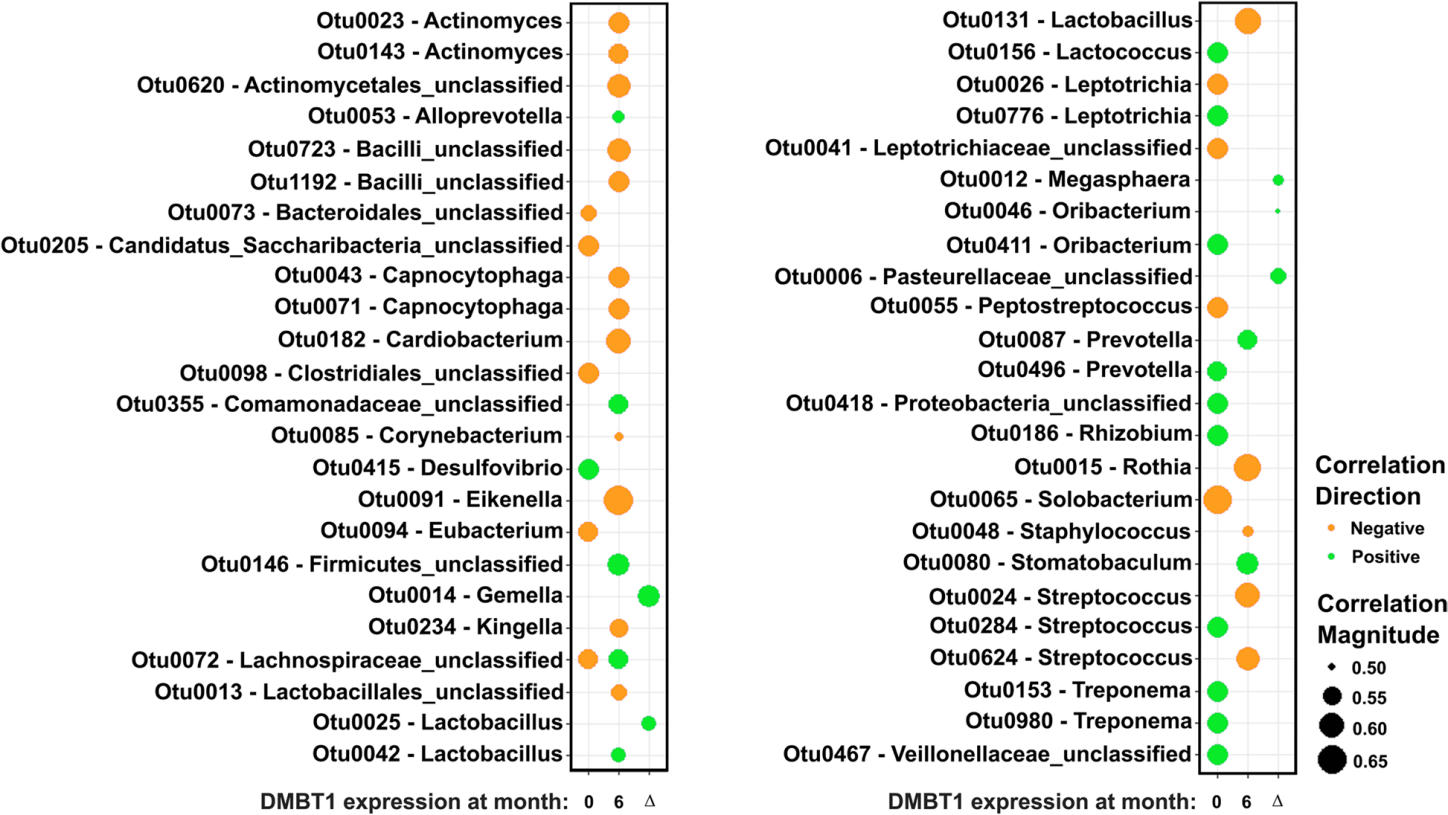
Downregulation of DMBT1 in saliva is associated with microbiome changes. Linear regression showing OTUs that correlate with DMBT1 expression at pre- (0 months), post-treatment (6 months), and difference in expression between post- to pre-treatment (Δ). Orange and green indicate negative and positive correlation directions, respectively. Circle size represents the correlation magnitude

In human saliva samples (Fig. 6), we applied a Pearson correlation model to investigate the relationship between DMBT1 protein expression with the composition of the salivary microbiome pre- and post-treatment (Fig. 8). Lower levels of DMBT1 at pre-treatment correlated with higher relative abundance of *Solobacterium* (OTU0065), an unclassified *Lachnospiraceae* (OTU0072), and an unclassified *Candidatus Saccharibacteria *spp. (OTU0205) and lower relative abundance of *Treponema* (OTU0153 and OTU0980), *Streptococcus* (OTU0284), and *Prevotella* (OTU496). Post-treatment, high DMBT1 levels negatively correlated with the abundance of *Actinomyces* (OTU0023 and OTU0143), *Eikenella* (OTU0091), *Capnocytophaga* (OTU0043 and OTU0071)*, Lactobacillus* (OTU0131)*,* and *Streptococcus* (OTU0024 and OTU0624) whereas there was a positive correlation between DMBT1 expression and the abundance of an unclassified Firmicutes member (OTU0146), unclassified *Comamonadaceae* (OTU0355), unclassified *Lachnospiraceae* (OTU0072), *Prevotella* (OTU0087), and *Stomatobaculum* (OTU0080) (Fig. 8). We also analyzed correlations between changes in DMBT1 expression with time and changes in OTU abundance. Interestingly, increased DMBT1 expression in saliva after treatment correlated with an increase in the abundance of *Gemella* (OTU0014), which was also enriched in responders to chemoradiotherapy at 6 months (Fig. 5G), unclassified *Pasteurellaceae *spp. (OTU0006), enriched in responders at 0 months, *Lactobacillus* (OTU0025), Megasphaera (OTU0012), and *Oribacterium* (OTU0046) (Fig. 8).

Together these findings show that changes in the oral microbiome in patients with SCC are associated with changes in the expression of the salivary anti-microbial protein DMBT1.

## Discussion

This is the first longitudinal study to investigate treatment-associated changes in the salivary microbiome in patients with SCC and associate these findings with changes in the expression of an anti-microbial protein in the saliva. Patients treated for SCC are at continued risk for recurrent or new tumors. Consequently, these patients are monitored for prolonged periods. Unfortunately, some of the treatment-induced changes in the oral mucosa are clinically indistinguishable from erythroplakias, a clinical phenotype of SCC. Repeated biopsies are not a feasible option. Consequently, saliva is very appealing in the pursuit of prognostic biomarkers because it is non-invasive, and sampling and processing are simpler compared to biopsy and histopathology. Saliva is comprised of a variety of factors that could give rise to prognostic biomarkers, including metabolites, nucleic acids, hormones, antibodies, growth factors, antimicrobial factors, and other proteins [54]. Notably, saliva allows for sampling of the oral microbiome. As the composition of the oral microbiota can vary by site within the oral cavity [55], the salivary microbiome may provide a good representation of microbial populations found throughout the oral cavity. Therefore, saliva collection is a potentially valuable tool in identifying microbial biomarkers of SCC and treatment responses.

SCC develops when transformed cells from pre-cancerous epithelium destroy the basement membrane and invade the underlying stroma, from where these cells can spread to adjacent and distant sites [56]. The microenvironment modulates both SCC progression and treatment resistance [57]. The salivary microbiome in SCC is poorly understood. Although multiple studies compared the oral microbiome in SCC with that in healthy or oral disease-related states and showed differences in microbial composition within the saliva, changes in the salivary microbiome of oral cancer patients with time and with treatment (chemoradiotherapy) are a more recent area of interest [42]. Longitudinal collection allows each individual to serve as their own control, thereby limiting variations due to inter-individual heterogeneity that would occur with a comparison of SCC and normal control samples. We observed an overall decrease in richness and modulation of specific bacterial populations with treatment, including an increase in *Lactobacillaceae* and *Bifidobacteriaceae* families, and a decrease in *Porphyromonadaceae* and *Prevotellaceae* post-treatment. Importantly, our study suggests that there are specific bacteria that either at the start or after completion of treatment are associated with response and recurrence. Moreover, a pilot proteomic analysis (data not shown) of saliva samples from our cohort of SCC patients pre- and post-treatment revealed significant upregulation of DMBT1 after treatment. In the present study, we validated this interesting finding and observed that the increase in salivary DMBT1 was correlated with a rise in certain bacterial populations within the saliva, including *Gemella*,* Lactobacillus*,* Megasphaera*, and* Oribacterium*. Our study showing microbiome changes between pre- and post-treatment saliva from patients with SCC supports the development of oral microbiome markers of SCC to assess response to treatment.

Bacterial sequencing studies have revealed that certain cancers are associated with dysbiosis, an imbalance in microbial diversity and community stability [3]. Studies using germ-free mice transplanted with cancer-associated gut microbiotas have provided evidence that dysbiosis can directly contribute to the development of cancer [58]. Microbial-induced mechanisms are consistent with the hallmarks of cancer [59] and include tumor-promoting inflammation, immune evasion, proliferative signaling, and genome instability [1]. The impact of the salivary microbiome on the pathogenesis of SCC is relatively under-explored. Studies comparing microbial composition in the saliva of patients with SCC have generally been small, but have nonetheless demonstrated differences between healthy and SCC-associated microbiotas [40]. Although a consistent SCC-associated microbial signature has not been identified likely due to small sample sizes and heterogeneity of patient populations, these studies have shown increased levels of bacteria belonging to *Prevotella*,* Fusobacterium*, and *Porphyromonas* in the SCC group [49]. Differences in microbial composition have also been demonstrated by analyzing SCC versus contralateral normal tissue sections from the same patient using a paired approach [60]. In both discovery and validation cohorts, there was a decrease in *Streptococcus* and *Rothia* species in cancer tissue compared to the contralateral normal control. Shin et al. (2017) compared primary tumor tissue, metastatic tissue, and normal tissue within the same patient and found similar results, notably an increase in *Fusobacterium* and a decrease in *Streptococcus* in cancer versus control [61]. In a cohort of patients with tongue SCC, Michikawa et al. showed that Fusobacterium is increased at the tumor site compared to adjacent normal tissue which had high Streptococcus and Rothia [62]. However, whether these changes directly contribute to the development of SCC is unknown. Besides the use of gnotobiotic models to demonstrate causality, normalization of the microbiota with curative treatments would be suggestive of a role for dysbiosis in disease pathogenesis. Guerrero-Preston et al. compared the salivary microbiome composition between SCC patients before and after surgical resection [63]. In this longitudinal study on 11 patients, they noted a reduction in alpha diversity (species richness) after surgery, but an increase in alpha diversity in patients with tumor recurrence. *Lactobacillus* and *Veillonella* increased after treatment although the small sample size precluded definitive conclusions. In contrast, a recent study showed a decrease in alpha diversity after treatment [40].

In the present study, we were able to analyze changes in the composition of the salivary microbiota between 0 and 6 months. Besides a reduction in overall richness, we observed notable increases in relative abundance in the families *Lactobacillaceae* and *Bifidobacteriaceae* and decreased abundance of families including *Porphyromonadaceae*,* Prevotellaceae*,* Neisseriaceae*, and* Leptotrichiaceae.* Some of these changes are consistent with other studies showing the enrichment of *Porphyromonadaceae*, *Prevotella*, and *Fusobacteria* and the depletion of *Neisseriaceae* in oral cancers [64–67]. Whether these bacterial populations are involved in cancer progression or maintenance of oral health remains to be determined.

Some commensal microbes affect the efficacy of chemotherapy. For example, *E. coli* interferes with the efficacy of gemcitabine and CB1954, inducing tumor resistance and cytotoxicity, respectively [68]. *Gammaproteobacteria* in human pancreatic ductal adenocarcinoma can metabolize gemcitabine, conferring tumor resistance to treatment that can be reverted with antibiotic therapy [68]. In contrast, drugs like cyclophosphamide and oxaliplatin have decreased efficacy in germ-free or antibiotic-mediated microbiome-depleted mice [69]. How commensal bacteria regulate treatment responses remains to be fully elucidated, but includes upregulation of cytokines with anti-tumor activity in myeloid-derived cells and promotion of CD8 T cell infiltration and activation [11, 70]. Definitive chemoradiotherapy is typically used in the treatment of locally advanced oral cancers with curative intent although recurrences are common. Thus, identifying biomarkers either prior to therapy or after completion of therapy that are predictive of response can have significant clinical implications and can be used to guide treatment. In the current study, at baseline prior to therapy and 6 months after chemoradiation, there was a significantly different microbiome composition between responders and non-responders, but no significant variation in diversity or richness. We also found OTUs at the start and end of treatment that correlated with response or recurrence. Interestingly, we identified an OTU within the genera *Prevotella* (OTU0029) that was associated with recurrence (i.e., non-responders) at the start of treatment and depleted after cancer therapy at 6 months, suggesting a potential role for this bacterial population in tumor progression. However, it should be noted that many of the OTUs identified as significantly different by LEfSe analysis were not consistently identified using ALDex2, which may be largely due to the differences in data pre-processing, normalization, and testing methods used in different methods [71]. Additional studies with a larger cohort of patients will be needed to evaluate microbial biomarkers within the saliva with prognostic significance.

Functional prediction of pathways that may be differentially represented by salivary communities before and after treatment revealed that microbial functions involved in lipopolysaccharide biosynthesis were enriched before treatment. Interestingly, two OTUs (*Leptotrichia* OTU0026 and *Capnocytophaga* OTU 0043) were negatively correlated with DMBT1 expression and both were high before treatment in patients. Both belong to genera that have lipopolysaccharide activities, which can be associated with cancer detection or progression [72, 73]. Lipopolysaccharide also activates Toll-like receptor 4 (TLR4), which is overexpressed in oral cancers and has been linked to oral carcinogenesis [74]. PICRUSt analysis also correlated two pathways related to amino acid metabolism with treatment response at 6 months, namely, phenylalanine, tyrosine, and tryptophan biosynthesis as well as valine, leucine, and isoleucine biosynthesis. Interestingly, these pathways were also previously associated with healthy individuals when compared with head and neck cancer patients [75].

A possible limitation of our study is the lack of long-term follow-up data. Almost half of SCC recur within 5 years, but due to the low number of recurrences at the 6-month period when microbiome sequencing data were analyzed, the relationship between the microbiome and tumor recurrence could not be fully investigated. A limitation of 16S rRNA sequencing and analysis of OTUs is that species-level information is difficult to obtain. Future studies using shotgun metagenomic sequencing will be informative in determining specific bacterial species and functions that are associated with treatment responses.

DMBT1, also known as salivary agglutinin, binds and neutralizes bacteria and viruses and activates the complement system [76, 77]. DMBT1 is a glycoprotein with a significant role in mucosal immunity. Recently, our group demonstrated suppression of DMBT1 in SCC with a critical role in invasion [44, 45]. In the present study, DMBT1 expression is low in saliva from patients with SCC and increases after treatment. It is primarily expressed in the epithelium and in secretions such as saliva, although some studies report DMBT1 in non-mucosal tissues including tooth surfaces [43, 78–80]. Given the variability in DMBT1 localization, it is possible that in different locations (soluble versus membrane), DMBT1 has specific functions.

The change in expression of salivary DMBT1 with accompanying changes in the microbiome suggests the possibility that DMBT1 may be important in modulating the oral microbiome and/or the tumor microenvironment to maintain homeostasis and resist carcinogenesis and tumor progression.

## Conclusion

This is the first longitudinal study to investigate treatment-associated differences in the oral microbiome in patients with SCC and associate them with changes in the expression of DMBT1. Our findings support the development of salivary biomarkers of SCC and microbiome biomarkers to predict response to treatment. Future studies should be directed toward candidate microbial species that are associated with response, such as *Gemella *spp. and *Leptotrichia* spp., including characterization of their effects in response to treatment in SCC and identification of underlying mechanisms.

## Methods

### Patient population and sample collection

Saliva samples were obtained from the University of Michigan Head and Neck Cancer Specialized Program of Research Excellence/Head and Neck Oncology Program (HNSPORE/HNOP) prospective epidemiology project. IRB approval and patient consent were obtained prior to saliva collection. Amongst 109 patients with SCC used for microbiome analysis, 50 patients were treated with chemoradiotherapy with available time 0 samples and 33 of those had available paired samples (baseline and 6 months post-treatment). Fifteen surgically treated patients had paired samples for pre- and post-treatment microbiome analysis (Table 1). The disease site in the chemoradiotherapy population (responders and non-responders) was primarily the oropharynx (38) but included the larynx (10), oral cavity (1), and unknown (1) (Table 2). Whole stimulated saliva was collected pre- and post-treatment (6 and 12 months post-diagnosis) for 5 min, as described [37]. The total volume was quantified, and the flow rate (ml/minute) was calculated. The samples were centrifuged, and protease and phosphatase inhibitors (1e^−4^ U/ml aprotinin, 1.2 mM Na_3_VO_4_ [sodium orthovanadate], 0.1 mg/ml PMSF [phenylmethylsulfonyl fluoride]) were added to the supernatant, which was aliquoted and frozen at − 80 °C. A schematic overview of the entire study including sample collection and analysis is shown in Fig. 1A.

### DNA isolation and amplification

DNA was isolated from saliva with a MagAttract PowerMicrobiome DNA/RNA Kit (Qiagen) using an epMotion 5075 liquid handling system. The V4 region of the 16S rRNA gene was amplified and sequenced with an Illumina MiSeq using MiSeq Reagent Kit V2 500 cycles (Illumina cat# MS102-2003), as described previously [81], and 3 µl of DNA (undiluted) was used for each standard PCR.

### Sequence processing and analysis overview

The 16S rRNA gene sequence data was processed and analyzed using the software package mothur (v.1.42.3) [82]. After sequence processing [83] and alignment to the SILVA reference alignment (release 132) [84], sequences were binned into operational taxonomic units (OTUs) based on 97% sequence similarity using the OptiClust method [85]. Processing and analysis steps are available in the mothur batch files (Supplement data dsilva2.sop1.batch and dsilva2.sop2.batch). Records of all steps performed in mothur are available in the mothur logfiles (Supplement data mothur.1582056286.logfile and mothur.1582305303.logfile). By calculating θ_YC_ distances (a metric that takes the relative abundance of both shared and non-shared OTUs into consideration) [86] between communities and using analysis of molecular variance (AMOVA) [87], we tested for statistically significant differences between the microbiota of different groups. NMDS was used to visualize θ_YC_ distances between samples. Linear discriminant analysis (LDA) effect size (LEfSe) and a Dirichlet-multinomial model after log-ratio transformation (ALDEx2) [48], a compositional differential abundance analysis tool, were used to determine if specific OTUs were differentially abundant in different groups [88]. Welch’s *t* test and Benjamini–Hochberg adjusted *p* values were used for ALDEx2 results. The taxonomic composition of bacterial communities was determined by classifying sequences within mothur using a modified version of the Ribosomal Database Project (RDP) training set (version 16) [89, 90].

Unpaired t tests were used for the comparison between all samples at 0 month and 6 months and between responders and non-responders at 0 month or 6 months. Paired *t* tests were used for the comparison between 0-month and 6-month samples for either chemoradiotherapy or surgery. We also investigated the diversity metrics, including the inverse Simpson index which was calculated. Predictive functional profiling of microbial communities using 16S rRNA sequences was performed using PICRUSt analysis [91], and pathways were identified using the KEGG classification.

### Salivary DMBT1 protein quantification

DMBT1 quantification was performed if the baseline and corresponding 6 and/or 12 months of samples were available from the same patient (Table 3). DMBT1 in saliva samples was quantified by immunoblot (WB) and enzyme-linked immunosorbent assay (ELISA). For immunoblot analysis, equal volumes of saliva were electrophoresed on 4–12% of Tris–glycine gels, and results were normalized by a sample volume. After transfer, DMBT1 was detected with goat anti-mouse DMBT1 (AF5915 R&D Biosystems).

For ELISA, saliva samples were diluted 1:2 in PBS and analyzed in duplicate using a DMBT1 ELISA assay (EKU03679 Biomatik, Wilmington, Delaware). Only samples with sufficient volume were analyzed by ELISA, which required a larger sample volume than with immunoblotting. Consequently, ELISA was performed on saliva samples from 28 patients, a subset of the 48 patients used for immunoblot analysis. Changes from baseline to month 6 and month 12 were tested in a linear mixed model of log-transformed values assuming compound symmetric variance structure and fixed effect for timepoint (baseline, month 6, month 12) with baseline considered the reference.

### Microbiome and DMBT1 protein expression correlation analysis

Pearson correlation coefficients between relative abundance scores and DMBT1 protein expression levels were calculated for 20 subjects with available paired relative abundances and DMBT1 expression quantifications (Table 4).

**Table 4:** Demographics distribution of patients used in microbiome and DMBT1 change correlation (Fig. 8)

**Variable**		**Up (*n* = 15)**	**Down (*n* = 5)**	
		*N* or mean (std)	*N* or mean (std)	***p* value**
**Age**	Years	57.1 (7.2)	59.2 (9.9)	0.62
**Gender**	Male	12	2	0.27
	Female	3	3	
**Clinical stage**	2	1	-	1.00
	3	2	1	
	4	12	4	
**T stage**	T1	2	1	0.67
	T2	8	1	
	T3	2	2	
	T4	3	1	
**N stage**	N0	1	2	0.17
	N1	3	0	
	N2	11	3	
**Disease site**	Larynx	1	1	0.25
	Oral cavity	2	-	
	Oropharynx	12	3	
	Nasopharynx	0	1	
**Treatment**	Chemotherapy	0	1	0.15
	Surgery	3	-	
	Chemoradiotherapy	12	4	
**ACE comorbidities score**	None	5	1	0.36
	Mild	8	3	
	Moderate	2	-	
	Severe	0	1	
**BMI**	Normal (15.5–24.9)	2	-	0.84
	Overweight (25–29.9)	6	3	
	Obese (30 +)	7	2	
**HPV status**	Negative	3	3	0.25
	Positive	12	2	
**Drinker**	Never	1	-	1.00
	Current	13	4	
	Former (quit > 12 months)	1	1	
**Smoker (cigarettes)**	Never	5	-	0.04
	Current	7	1	
	Former (quit > 12 months)	3	4	

### Mouse saliva studies

UM-SCC-1 cells (8 × 10^5^) in matrigel or matrigel alone were injected subcutaneously in athymic nude mice (Ncr-nu/nu, age 6 weeks, *n* = 15 for the tumor group, and *n* = 13 for the control group) according to the protocol approved by The University of Michigan Institutional Animal Care and Use Committee (IACUC). Enlarging tumors were monitored and measured at least 3 times per week. Saliva was collected from adult mice at 11 weeks (S1) and at 16 weeks or prior to euthanasia (S2), according to the approved protocol. Pilocarpine was injected intraperitoneally (IP) at a dose of 20 µg/20 g body weight. Stimulated whole saliva was collected (Fig. 7A). The volume of each saliva sample was quantified; samples were centrifuged at 10,000 rpm for 20 min at 4 °C. After centrifugation, saliva samples were transferred to new tubes, protease inhibitors were added, and samples were stored at − 80 °C. DMBT1 quantification by immunoblot was performed using the same protocol used for human saliva samples. A paired t-test was used to test for significant change over time within groups and a *p*-value <0.05 was considered significant. A linear mixed effects model with interaction term for group x time assuming compound symmetric variance structure within subject, was used to further test for difference in slope.

### Supplementary Information

**Additional file 1: Fig. S1. **(A) Relative abundance of OTUs between salivary microbiomes at 0 (pre-treatment) and 6 months (post-treatment) for SCC. (B) Most differentially abundant OTUs before and after treatment based on LEfSe analysis (LDA cutoff of 3). **Fig. S2.** Most differentially abundant OTUs between salivary microbiomes before (0 months) and after chemoradiotherapy (6 months) based on LEfSe analysis (LDA cutoff of 3).

## Data Availability

The sequence reads of data presented in this manuscript are deposited in the Sequence Read Archive database under the project PRJNA906710, samples SAMN31934173 to SAMN31934357.
