# Supplementary figures and images for: Salivary microbiome changes distinguish response to chemoradiotherapy in patients with oral cancer
Source: Microbiome. 2023 Nov 30;11:268. doi: 10.1186/s40168-023-01677-w (PMC10687843; doi:10.1186/s40168-023-01677-w)

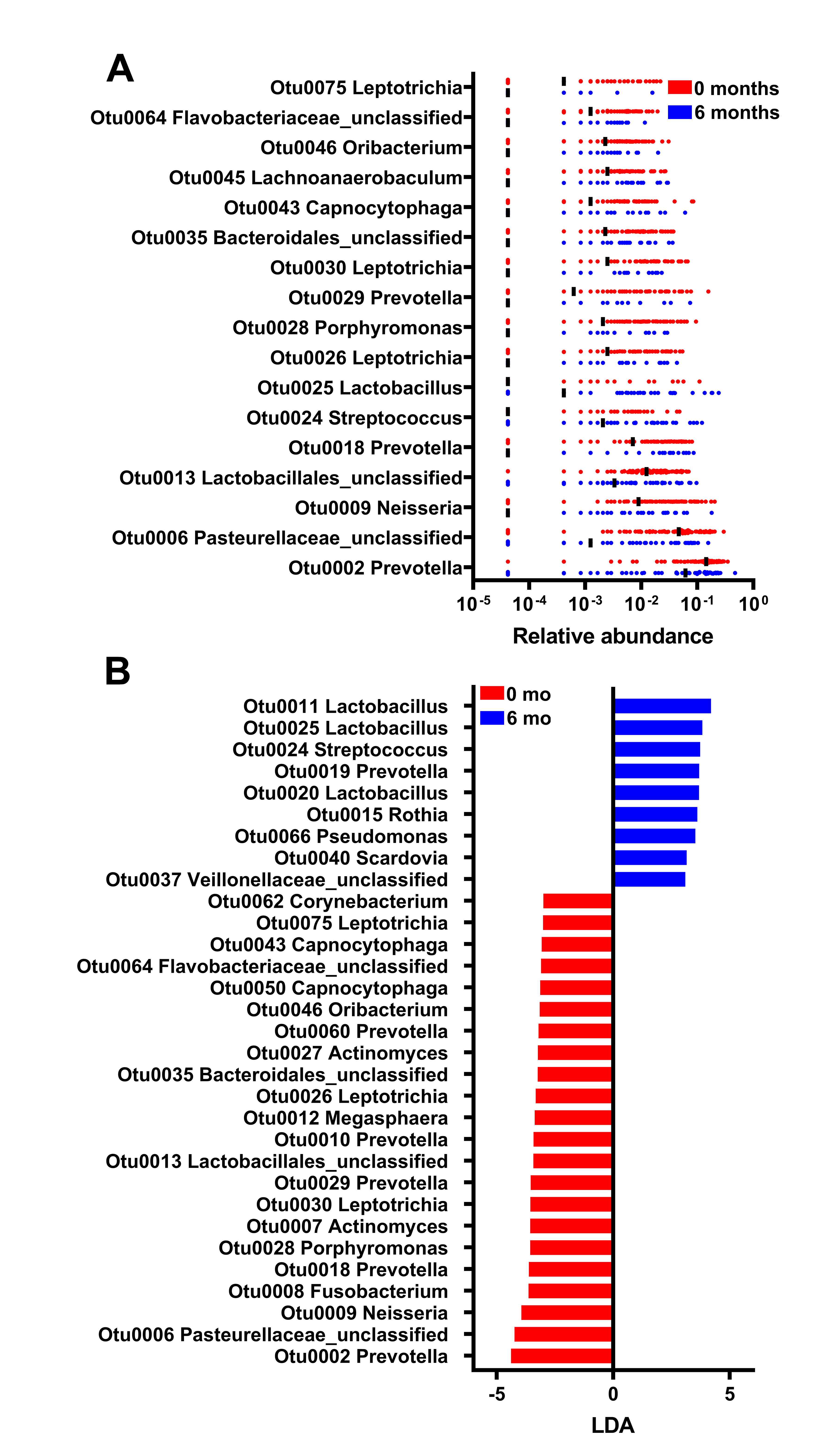

Supplement: Supplementary file 2 — Additional file 1: Fig. S1. (A) Relative abundance of OTUs between salivary microbiomes at 0 (pre-treatment) and 6 months (post-treatment) for SCC. (B) Most differentially abundant OTUs before and after treatment based on LEfSe analysis (LDA cutoff of 3). Fig. S2. Most differentially abundant OTUs between salivary microbiomes before (0 months) and after chemoradiotherapy (6 months) based on LEfSe analysis (LDA cutoff of 3). [file 40168_2023_1677_MOESM1_ESM.zip › Supplemental Figure 1.tif]

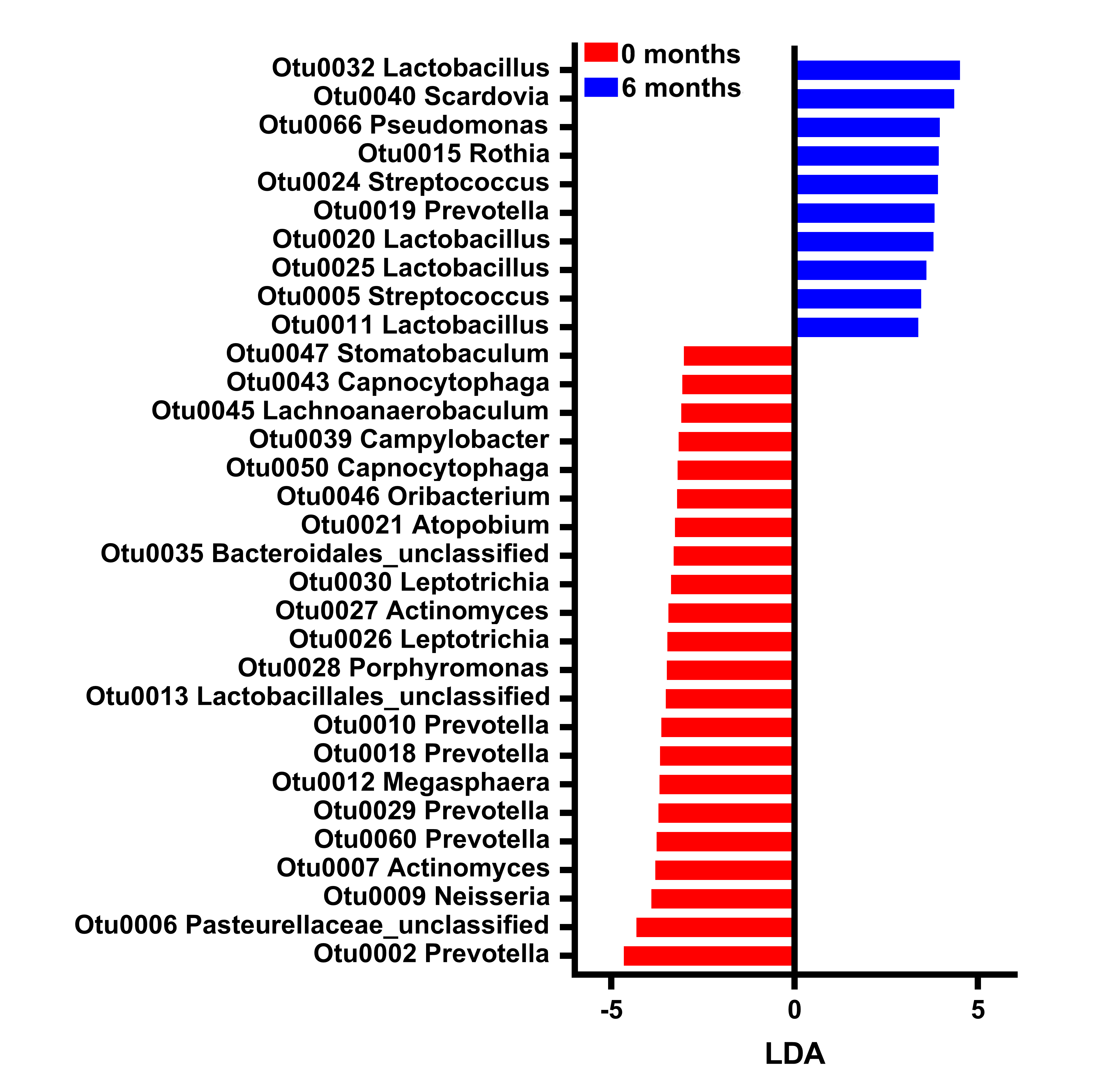

Supplement: Supplementary file 2 — Additional file 1: Fig. S1. (A) Relative abundance of OTUs between salivary microbiomes at 0 (pre-treatment) and 6 months (post-treatment) for SCC. (B) Most differentially abundant OTUs before and after treatment based on LEfSe analysis (LDA cutoff of 3). Fig. S2. Most differentially abundant OTUs between salivary microbiomes before (0 months) and after chemoradiotherapy (6 months) based on LEfSe analysis (LDA cutoff of 3). [file 40168_2023_1677_MOESM1_ESM.zip › Supplemental Figure 2.tif]
